# Ultrastructural analysis of synapses and mitochondria in the hippocampus of depressed patiens

**DOI:** 10.1192/j.eurpsy.2024.747

**Published:** 2024-08-27

**Authors:** A. Sebők-Tornai, D. Csabai, C. Szekeres-Paraczky, Z. Maglóczky, M. Simon, C. A. Stockmeier, B. Czéh

**Affiliations:** ^1^Szentágothai Research Centre, University of Pécs; ^2^Laboratory Medicine, University of Pécs, Medical School, Pécs; ^3^Human Brain Research Laboratory, Inst. Experimental Medicine, ELKH, Budapest; ^4^Psychiatry and Psychotherapy, University of Pécs, Medical School, Pécs, Hungary; ^5^Psychiatry and Human Behavior, University of Mississippi Medical Center, Jackson (MS), United States

## Abstract

**Introduction:**

Major depressive disorder (MDD) is a common multifactorial disorder, but the exact pathophysiology is still unknown. in vivo and post-mortem studies document volumetric and cellular changes in the hippocampus of depressed patients. Chemical synapses are key functional units of the central nervous system and earlier studies found reduced number of synapses in the prefrontal cortex of depressed patients (Kang HJ *et al*. Nature Medicine 2012;18(9):1413-1417). Mitochondria are intracellular powerhouses generating chemical energy for cellular biochemical reactions. Recent findings suggest that individuals with impaired mitochondrial function may be vulnerable to develop psychopathologies.

**Objectives:**

We investigated synapses and mitochondria in post-mortem hippocampal samples from psychiatric patients.

**Methods:**

The three study groups were: 1) MDD patients (n=11); 2) patients with alcohol dependence (n=8) and 3) controls (n=10). Controls were individuals who accidentally deceased and had no neuropsychiatric disorders. Three sub-regions of the hippocampus (dentate gyrus, CA3 and CA1 areas were investigated. Ultrathin sections were examined, and photomicrographs were taken for further analysis using a JEOL JEM 1400 FLASH transmission electron microscope. Systematic quantitative analysis was conducted with the Neurolucida system using unbiased counting principles.

**Results:**

We could not detect any differences in synapse and mitochondria densities between the patients and controls subjects.

**Conclusions:**

Our preliminary data suggest that despite our expectations hippocampal synapse and mitochondrial densities are rather constant parameters which are not easily affected by psychopathology or alcohol consumption. Potential methodical limitations may also explain this negative finding.

FUNDING:

This research was founded by the Hungarian Brain Research Program 3 and by the TKP2021-EGA-16 project. A.S.T. was supported by the ÚNKP-23-3-I New National Excellence program of the Ministry for Culture and Innovation from the source of the National Research, Development and Innovation Fund.

**Disclosure of Interest:**

None Declared

